# Editorial

**DOI:** 10.1007/s00414-022-02892-0

**Published:** 2022-09-24

**Authors:** Ingo Wirth, Tony Fracasso, Heidi Pfeiffer, Andreas Schmeling

**Affiliations:** 1 Berlin, Germany; 2 University Center of Legal Medicine (CURML), Geneva University Hospital, Geneva, Switzerland; 3 Institute of Legal Medicine, University Hospital Münster, Münster, Germany

100 years ago, Berlin publisher Julius Springer released the first volume of the *Deutsche Zeitschrift für die gesamte gerichtliche Medizin* (*German Journal of Entire Legal Medicine*). With the exception of a war-related interregnum from 1944 to 1948, the broadly scoped journal was published with this title until 1969. In 1970, responding to changes in the designation commonly used to describe the specialty and institutes of legal medicine, it became the *Zeitschrift für Rechtsmedizin* (*Journal of Legal Medicine*). In renaming it the *International Journal of Legal Medicine* in 1991, the publishing house and editor were following the growing trend towards academic internationalisation. Under the editorship of Bernd Brinkmann, *International Journal of Legal Medicine* has become the world’s leading journal of legal medicine and has maintained this top position ever since.

The 135 volumes published to date reflect advances in legal medicine over the past 100 years. On the occasion of the anniversary, the amazing development of the journal will be honored in a four-part series of articles. Part 1 covers the period from 1922 to 1944, Part 2 from 1948 to 1969, Part 3 from 1970 to 1990, and Part 4 from 1991 to 2022. The articles analyze the major areas of focus of the journal during the respective period and place it in the context of the development of our discipline.

Part 1 of the article series appears in this issue. We wish you interesting reading.

